# Effects of Virtual Reality Exercises versus Isokinetic Exercises in comparison with Conventional Exercises on the Imaging Findings and Inflammatory Biomarker Changes in Soccer Players with Non-Specific Low Back Pain: A Randomized Controlled Trial

**DOI:** 10.3390/ijerph20010524

**Published:** 2022-12-28

**Authors:** Gopal Nambi, Mshari Alghadier, Faizan Zaffar Kashoo, Osama R. Aldhafian, Naif A. Nwihadh, Ayman K. Saleh, Mohamed A. Omar, Tohamy G. T. Hassan, Mohamed Nagah Ahmed Ibrahim, Hassan Fathy El Behairy, Abdehamid A. Attallah, Mohammed Abdelgwad Ismail

**Affiliations:** 1 Department of Health and Rehabilitation Sciences, College of Applied Medical Sciences, Prince Sattam bin Abdulaziz University, Al-Kharj 11942, Saudi Arabia; 2 Department of Physical Therapy and Health Rehabilitation, College of Applied Medical Sciences, Majmaah University, Al Majmaah 11952, Saudi Arabia; 3 Department of Surgery, College of Medicine, Prince Sattam bin Abdulaziz University, Al-Kharj 11942, Saudi Arabia; 4 King Fahad Medical City, Riyadh 11525, Saudi Arabia; 5 Department of Orthopedic, Faculty of Medicine for Girls, Al Azhar University, Cairo 11651, Egypt

**Keywords:** chronic non-specific low back pain, exercise, imaging findings, inflammatory biomarkers, soccer players

## Abstract

Chronic non-specific low back pain (CNLBP) is the most common musculoskeletal problem. The purpose of this study was to investigate the effects of advanced physiotherapeutic exercise programs on imaging findings and inflammatory biomarkers in soccer players with CNLBP. In total, 60 CNLBP participants were divided into virtual reality exercise (VRE; *n* = 20), isokinetic exercise (IKE; *n* = 20), and conventional exercise (*n* = 20) groups. Pain intensity, imaging findings (muscle cross-sectional area (CSA) and muscle thickness), and changes in inflammatory biomarkers (CRP, TNF-α, IL-2, IL-4, and IL-6) were measured at baseline and after four weeks. After four weeks of intervention, there was a significant improvement (*p* = 0.001) in pain intensity for the VRE vs. IKE (0.7; CI 95% 0.38 to 1.07) and VRE vs. conventional (3.0 CI 95% 2.68 to 3.31) groups. The IKE group showed a greater number of significant changes in muscle CSA and muscle thickness than the other two groups (*p* < 0.001). Moreover, the VRE group showed significant improvement in inflammatory biomarker measures compared with the other two groups (*p* < 0.001). In CNLBP, virtual and isokinetic exercises had equal effects on reducing pain intensity. Isokinetic exercise is beneficial in increasing the muscle CSA and thickness, and virtual exercises are helpful for attenuating the inflammation process in soccer players with CNLBP.

## 1. Introduction

Soccer is one of the world’s most popular team sports, with over 265 million people globally playing this sport for recreation or on a professional level. With the increasing popularity of this sport, there has been a rise in the number of associated injuries [1]. Low back pain (LBP) and lower limb injuries are among the injuries most commonly reported in elite soccer players [2]. In addition, players of sports in other categories such as skiing, rowing, golf, volleyball, track and field, swimming, and gymnastics are also at greater risk of suffering from LBP. Incidence rates of up to 30% LBP have been reported in athletes depending on the specific sport in which they are involved [3].

LBP is usually defined as pain and discomfort localized below the costal margin and above the inferior gluteal folds, with or without leg pain. Chronic non-specific low back pain (CNLBP) is described as continual pain for 12 weeks or more without specific known pathology [4]. Stability of the lumbar spine plays a critical role in preventing and reducing the risk of LBP-related injury, and the importance of core muscle function and coordination has been highlighted in several biomechanical studies [2]. Trauma to soft tissues of the lower back region during sporting activity can affect core muscle action and lead to muscle imbalance [5]. Alterations in core muscle function can lead to a wide range of atypical movements, leading to low back pain in the future [6]. Improper activities during the training session may also be a cause of abnormal core muscle activity, which may further aggravate the condition. Therefore, different intervention and training protocols were framed to prevent such sports injuries, and, in addition, post-injury training sessions have been framed to manage these conditions [7,8,9]. Trained team coaches and sports physiotherapists employ these training sessions to prevent and treat such injuries [10].

Recently, Nambi et al. observed that advanced exercise procedures have succeeded conventional techniques such as spinal manipulation, laser therapy, and therapeutic exercises, with these new advanced exercise procedures showing positive results [11]. Virtual reality exercise (VRE) is a popular hi-tech exercise procedure used in hospital setup, where a computer software program generates a virtual environment. Such a training procedure is usually used to treat balance disorders in patients with neurological disorders. At the same time, there are inadequate data on sports rehabilitation, especially regarding its effects on musculoskeletal conditions such as low back pain. The principle on which virtual training works is that it activates audiovisual cues by playing virtual games. Moreover, virtual exercise improves the functional capacity of an individual by selecting an activity within their capability [12,13]. It makes the training more enjoyable and eases training during rehabilitation, thereby offering a sense of security to the patients. The VRE technology stimulates sensory function through neural facilitation techniques, and these changes in the nervous system further trigger the muscles for new motor learning [14,15].

Clinical studies on chronic non-specific LBP have found that isokinetic exercise (IKE) has significant and consistent results on LBP, with a positive correlation between LBP and core muscle dysfunction [16,17]. These exercises are usually provided by isokinetic devices, which are used for training and rehabilitating different orthopedic conditions in a rehabilitation setup. The device itself creates different levels of resistance, which provides constant speed to the movement, no matter how much force is being applied by the patient. Studies report that these exercises improve the condition by consistently enhancing muscle strength all through the range of movement [18]. Despite these benefits, an experienced person is required for operating IKE, and a well-established setup is also needed to provide such treatment to patients; hence, there is a paucity of well-executed studies in this field. Moreover, exercise programs different to those of conventional strength exercise have proven to reduce pain intensity and improve the physical function of patients with CNLBP [19]. Krzysztofik et al. reported that resistance training is a primary exercise intervention used to develop muscle strength [20].

Imaging tools such as magnetic resonance imaging (MRI) and ultrasound (US) are commonly used to evaluate the muscle cross-sectional area and muscle thickness, respectively, in clinical settings [2,21,22]. A study by Lee et al. stated that MRI of the lower lumbar level muscle cross-sectional area (CSA) at the L5 level can be considered a prognostic factor concerning CLBP chronicity [21]. Furthermore, ultrasound is a more reliable and valid tool to measure multifidus muscle thickness for older adults with CLBP, but has limitations for measuring intramuscular fat in the aging population [22]. Klyne et al. observed that different training methods increase various antagonistic mediators such as C-reactive protein (CRP) and pro-inflammatory cytokines such as tumor necrosis factor alpha (TNF-α) and anabolic components such as interleukin IL-1β and 6 [23].

There are studies that have investigated the short- and long-term effects of virtual reality, isokinetic, and conventional exercises on different outcome measures such as pain intensity, muscle strength, range of motion, functional disability, kinesiophobia status, sleep quality, and health-related quality of life [12,13,17,18,19,20]. There are, however, no studies that have identified the immediate effects of these exercise methods on the imaging findings and inflammatory biomarker changes in soccer players with chronic non-specific low back pain. Therefore, the objective of this trial was to investigate the imaging findings and inflammatory biomarker changes in soccer players with chronic non-specific low back pain after participating in virtual exercise, isokinetic exercise, and conventional exercise. The study hypothesis is that there is no differences between these exercises in terms of imaging findings and inflammatory biomarker changes in soccer players with chronic non-specific low back pain.

## 2. Methods

### 2.1. Study Design

This clinical trial was a prospective, randomized, single-blinded, parallel-arm study where the subjects were distributed equally into three groups. The research received approval from the Department Ethical Committee (DEC), Prince Sattam Bin Abdulaziz University, Saudi Arabia, and was given the referral number RHPT/019/046. The study was executed in consensus with the Declaration of Helsinki recommendations. The study was retrospectively registered in the clinical trial registry with the registration number NCT05253599 on 24 February 2022. The trial was executed as per the CONSORT guidelines and was conducted between January 2020 and December 2021. The trial was conducted in the Department of Physiotherapy, Prince Sattam bin Abdulaziz University, Saudi Arabia, and the participants were enlisted from the University Hospital and King Khalid Hospital, Riyadh, Saudi Arabia. Before the start of the trial, all participants were informed regarding information about the risks and benefits of the treatment modalities in this study, and they read and gave their consent before participation.

### 2.2. Participants

The participants were selected based on the following selection criteria: male soccer players in the age range of 18–25 years, chronic non-specific LBP for three or more months, and pain score ranging from 4 to 8 on a 10-centimetre visual analog scale. The exclusion criteria were lumbar stenosis, lumbar radiculopathy, lumbar spondylolisthesis, spinal injuries, associated low back muscle and tendon injuries, fracture of the pelvic bone and lower extremity bones, spine dysfunctions, awaiting spine surgery, participants taking steroids, medications or analgesics, having serious pathologies of the thoracolumbar spine, participating in other resistance training and physical training programs, and non-cooperativity.

### 2.3. Recruitment, Randomization, and Allocation

University-level soccer players with CNLBP were recruited by sending personal emails, in which every potential soccer player was given an equal opportunity to participate in the study. Using a three-block randomization method, 60 participants were assigned to the VRE (*n* = 20), IKE (*n* = 20), and conventional (*n* = 20) exercise groups. The participant allocation was conducted through sealed envelopes by a blinded therapist.

### 2.4. Blinding

The treating physiotherapist could not be blinded due to the method and research setup. The researcher who evaluated the study outcome measures was blinded. Patients were instructed not to reveal details of their treatment to their fellow members and the evaluating physiotherapist.

### 2.5. Interventions

**Figure 1:**
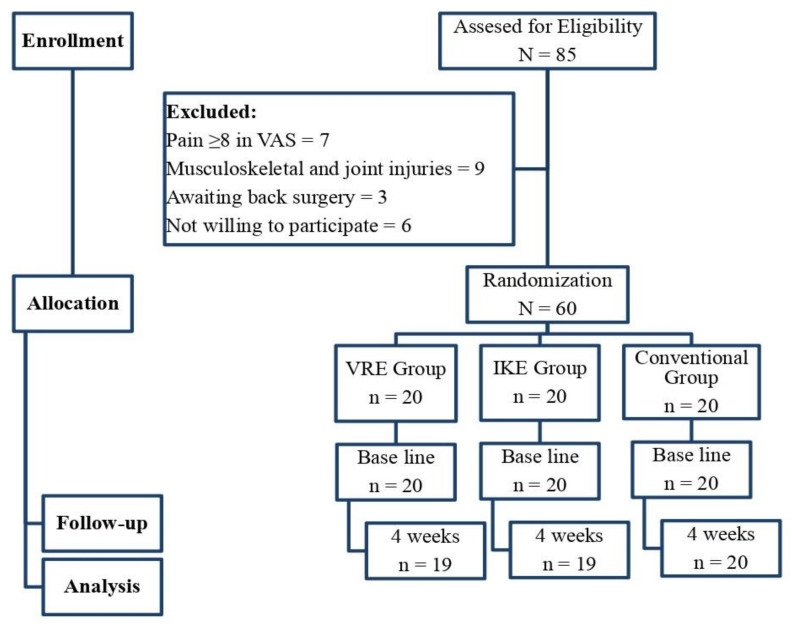
Flowchart showing the study details.

The exercise protocol, which lasted for four weeks, was conducted by a trained physical therapist with over 10 years of clinical practice. Seven patients with severe pain (score of higher than 8 in VAS), nine patients who had associated muscle, bone, and joint problems, three patients requiring surgeries, and six patients who did not consent to be a part of this study, were excluded from the trial (Figure 1).

**Figure 2:**
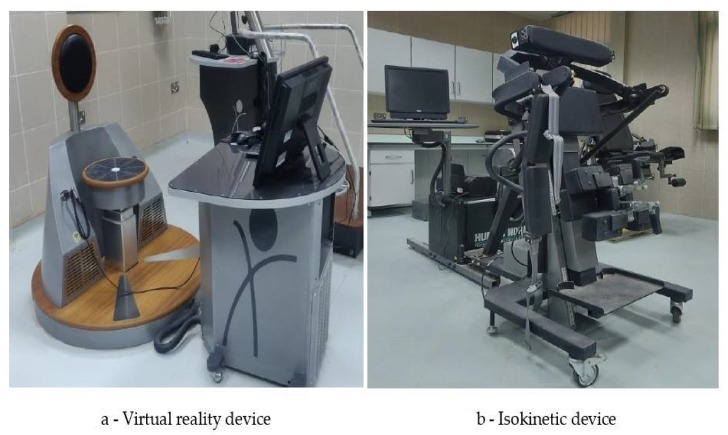
The virtual reality and isokinetic device.

The VRE group received virtual reality exercises with the device for this purpose (Figure 2a; Pro-Kin, Techno body, Italy), and the strength training focused on the trunk muscles of the back region. All participants in this group performed these exercises, and the subjects received individual demonstrations on how to play the VR games. The virtual training exercises were performed in the upright position, which offers resistance to the trunk movements. A car race game was chosen from the list of games, and training was given to focus on the back muscles. The participant was asked to sit on the moving game chair and instructed to watch the game on the desktop monitor. Joystick actions were performed by changing the trunk positions, such as moving backwards, forward, to the left or right, based on the position of the target on the display screen. During the training, the participants were permitted to move their trunk in all directions within their pain-free limit. Training progressed in a gradual manner, where movement slowly became more difficult and harder to perform, requiring the participant to use additional contractions dependent on the core stability muscles. The toughness of the race was increased by adjusting the number of opposing cars, the angle and steepness of the roads, and the quality of the terrain. Increasing the toughness of the race increased resistance to the movement [11,24].

For isokinetic exercise training, the participant was asked to stand in an upright position in the isokinetic device (Figure 2b; Humac Norm, Stoughton, USA). The knee joints were kept at 15° of flexion for relaxation of lower extremity muscles, and Velcro bands were fastened around the upper chest, pelvis, mid-thigh, and leg region to avoid accessory and unnecessary movements. The lower back region was kept free for a free range of movements with 10–15° of backward movement and 70–80° of forward movement. The fulcrum point of the isokinetic device was set at the lower back region, which is usually present 3–4 cm below the midpoint of the iliac crest of the pelvic bone in the lateral view. The moving arm was fixed to the patient’s trunk, and the resisting force was provided by the arm from the front and back of the trunk. To reduce the adverse consequences and risks, the required changes were implemented by following the manufacturing guidelines. The moving arm was able to move freely with the range of 10–15° of backward movement and 70–80° of forward movement, and 0^o^ was set as a neutral range. The participants were instructed to do these movements at an angular velocity of 45, 60, and 90°/s with 10 repetitions of three sets. Thirty seconds rest was given between each set, and sixty seconds rest was given between each pace. The trunk flexor and extensor muscles were targeted, and the peak torque was measured using an isokinetic dynamometer [18,25].

The conventional exercise group performed routine balance exercises to train the lower trunk muscles. The standardized conventional exercises actively involve the abdominal, deep abdominal, and back muscles. The participants performed 10–15 reps/day of these exercises, and the lower limb muscles were stretched for three repetitions, 15 s each, details of which are attached as a supplementary file (Appendix A). The virtual reality, isokinetic, and conventional exercises were performed continuously for thirty minutes per session, five days a week, for 4 weeks. All the participants in the three groups were instructed to refrain from other forms of physical and sports activities during this 4 week period. All the participants in the three groups also received 20 min sessions of hydro collateral packs and five minutes of continuous ultrasound with the parameters of 1 MHz frequency and 1.5 w/cm^2^ intensity at the lower back region for five days a week for 4 weeks [19].

### 2.6. Outcome Measures

Clinical measures such as maxVO_2_ and heart rate were measured for the ability of the participants to be involved in the intervention. For measuring maxVo_2,_ the participants were asked to run on a treadmill with a maximum limit, with a special face mask worn on the face to measure the amount of air breathed in and breathed out during the exercise. The outcome measurements were measured before the intervention (baseline) and 4 weeks after (post) the intervention.

#### 2.6.1. Primary Outcome

Pain Intensity: This was evaluated using the visual analog scale (VAS) of a 10 cm line, where the extreme left was inferred as “no pain” and the extreme right was inferred as “extreme pain”. The participants were asked to enter their level of pain intensity on the scale. It is a valid and reliable tool to measure pain intensity in CLBP patients [26].

#### 2.6.2. Secondary Outcome

Cross-sectional area (CSA): Cross-sectional magnetic resonance images (MRI) of psoas major, quadratus lumborum, multifidus, and erector spinae were recorded at the level of L4/L5. The CSA images were taken with an MRI device (Siemens, Munich, Germany) with the following parameters: T1 (TR/TE 411-610/12 msec, flip angle 150°) and T2-weighted (TR/TE 3230-5630/88-104 msec, flip angle 170°) axial turbo spin echo images; 4mm slice thickness; image resolution: 256 × 256 (T1), 320 × 320 (T2). The best image was selected by a radiologist, and the muscle boundaries manually outlined and included for analysis. Measuring the CSA of para-spinal muscles has good intra-rater reliability [21].

Muscle thickness: The right and left sides of the multifidus muscle thickness were measured in a resting state with an ultrasound (Hitachi, Tokyo, Japan) device. The participants were placed in the prone position and a pillow was kept under the abdomen to straighten the curve. The measurements were taken from the apex of the facet joint of L4, and on the junction between the thoracolumbar fascia and subcutaneous fat, and have good intra-rater and inter-rater reliability [27].

Inflammatory biomarkers: A 10 mL venous blood sample was collected from all the subjects by a trained phlebotomist in sterile P100 test tubes between 08:00 and 11:00. After initial inversion, the tubes were incubated without further agitation. After the incubation period, the tubes were processed by centrifugation at 2500 g for 20 min. Initially, the plasma was isolated from the blood samples and kept in the freezer at −70 degree Celsius. The levels of inflammatory biomarkers, such as C-reactive protein, tumor necrosis factor-α, and interleukins 2, 4, and 6, were measured using ELISA kits (R&D, Minneapolis, MN, USA), and the results were recorded. A systematic review by Morris P et al. stated that these pro-inflammatory biomarkers are altered in acute, subacute, and chronic non-specific low back pain [28].

### 2.7. Sample Size

The study predicted an intergroup difference of 1 point in pain intensity as measured by the visual analog scale, with an estimated standard deviation of 1.84 points. The following parameters were provided: 80% power, 5% alpha, and a maximum subsequent loss of up to 15%. Consequently, 60 subjects (20 for each group) were recruited for this study. The estimations used in the sample size calculations were less than the minimum clinically important differences recommended for improving the accuracy of the intervention efficacy estimates used in the computations [11].

### 2.8. Statistical Tests

Descriptive analyses were conducted to provide background information on the participants, and the normality of the data was checked using Kolmogorov–Smirnov test. Multivariate, mixed model, repeated measure (MMRM) analyses were conducted to examine relationships between the groups and the time effects for all the dependent variables. The mean ± standard deviation (SD) and 95% CI (upper–lower limit) for all the outcome variables are shown as error bars in the line graphs. To compare the differences between the experimental and control group in each variable, a one-way ANOVA test was used. To adjust for multiple comparisons, post hoc Tukey–Kramer tests were performed to compare absolute mean differences. The effect size, Cohen’s d, was calculated to assess the power of the study. A *p*-Value of less than 0.05 was considered to indicate a statistically significant difference. All analyses were performed using SAS statistical software (version 9.4, SAS Institute, Cary, NC, USA). The graphs were generated using GraphPad Prism version 9.12 for Windows (GraphPad Software, La Jolla California USA).

## 3. Results

### 3.1. Participants

Initially, 85 participants were evaluated for this study, and 25 were excluded, resulting in 60 participants being randomized into the three distinct groups for the study. The reports of the study were analyzed to treat assumptions. By the end of the study, one participant from the VRE and IKE groups did not complete the study due to personal inconveniences. At baseline, the descriptive demographic features of the study participants did not show any difference between the three groups. Clinical measures, such as maxVO_2_, heart rate, years of playing, and duration of injury, were measured for the ability of the participants to be involved in the intervention. The Kolmogorov–Smirnov test results indicate the normal distribution of the study samples between the groups (Table 1).

### 3.2. Pain Intensity

The mixed model with repeated measures (MMRM) for the group and time (3 × 2) for the pain intensity showed significant changes F (2, 57) = 295.6 (*p* = 0.001) between the three groups. After four weeks of different exercise protocols, there was a significant improvement in pain intensity level when comparing the VRE vs. IKE (0.7; CI 95% 0.38 to 1.07) and VRE vs. conventional (3.0 CI 95% 2.68 to 3.31) groups (*p* = 0.001). The same improvement could be seen for IKE vs. conventional (2.3; CI 95% 1.98 to 2.61) group (Table 2). The post hoc Tukey–Kramer test showed statistically significant (*p* = 0.001) changes in the VRE, IKE, and conventional groups. The standard mean difference (MCID = 3.0) showed statistically and clinically significant changes in then pain intensity level in the VRE group compared with the IKE and conventional groups. The effect size (d = 5.37) showed a greater effect toward the VRE group than the other two groups.

### 3.3. Radiological Measures

The baseline report on the cross-sectional area between the VRE, IKE, and conventional exercise groups showed no significant difference (*p* ≥ 0.05) in the tested back muscles. After four weeks of different exercise interventions, there was a statistically significant increase (*p* = 0.001) in cross-sectional area in the three groups. The post hoc Tukey–Kramer test results showed greater increase in the cross-sectional area of back muscles in the IKE group than the other two groups after four weeks of training (Table 2). The effect size (Cohen’s d) of cross-sectional area of right and left side of psoas major (d = 2.0, 1.71), quadratus lumborum (d = 1.14, 1.42), multifidus (d = 1.5, 1.11), and erector spinae (d = 0.66, 1.09) showed a larger effect toward the IKE group than the VRE and conventional groups. The overall clinical and statistical measures showed an advantage toward the IKE group over the VRE and conventional groups. Furthermore, there was greater evidence of an increase in muscle thickness in both the right and left side of the multifidus muscle in the IKE training than in VRE and conventional training based on the post hoc Tukey–Kramer test. The effect size (Cohen’s d) of muscle thickness of the right and left side of the multifidus muscle (d = 0.27, 0.22) showed a more moderate effect toward the IKE group than the VRE group.

### 3.4. Inflammatory Biomarker Measures

**Figure 3:**
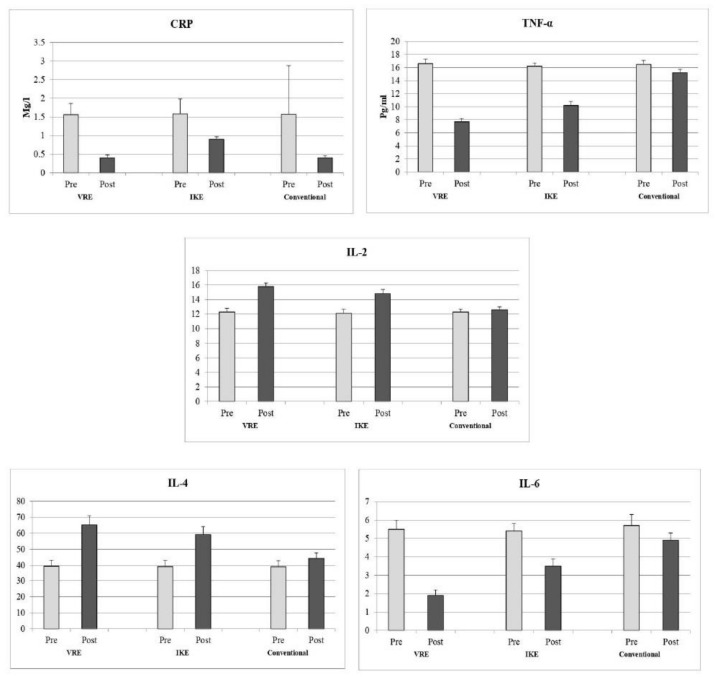
Pre and post analysis of inflammatory biomarkers of VRE, IKE, and conventional groups. CRP—C-reactive protein, TNF-α—tumor necrosing factor, IL—interleukin, VRE—virtual reality exercise, IKE—isokinetic exercise.

The baseline report on the inflammatory biomarkers C-reactive protein (CRP), tumor necrosis factor (TNF-α), and interleukins 2, 4, and 6 between the VRE, IKE, and conventional training groups showed no significance difference (*p* ≥ 0.05). After four weeks of different exercise interventions, there was a significant improvement in all the inflammatory biomarkers between the three groups (*p* = 0.001). The post hoc Tukey–Kramer test showed statistically significant changes in the VRE group to the other two groups after four weeks of training (Table 3). The effect size (Cohen’s d) of inflammatory variables of CRP (d = 6.66), TNF-α (d = 4.54), IL-2 (d = 1.81), IL-4 (d = 1.16), and IL-6 (d = 4.57) showed larger effect toward VRE group than the IKE group, which is shown in Figure 3.

## 4. Discussion

This randomized controlled trial evaluated the clinical differences in the intensity of pain, radiological changes in muscle cross-sectional area and thickness, and inflammatory changes in soccer players with chronic LBP before and after VRE, IKE, and conventional exercises. Before treatment, higher values of pain intensity in the patients were observed in the study data. After four weeks of different exercise intervention, there was a significant improvement in pain intensity level between the VRE and IKE groups with an effect size of d = 5.37, which is considered a large effect. The similar significant changes were noted in the secondary outcome measures such as for muscle cross-sectional area, muscle thickness, and inflammatory biomarkers. This observation complied with Treede et al., who concluded that high VAS scores are a result of injury to the lower back and its associated soft tissues [29]. An increase in pain intensity can progressively induce inhibition of the para-spinal muscle activity, which results in impaired core muscle function [30]. Gold et al. stated that VR-induced pain modulation originates from inter-cortical modulation among signaling pathways of the pain matrix through attention, emotion, memory, and other senses (e.g., touch, auditory, and visual), thereby producing analgesia. An overall decrease in the pain matrix may be accompanied by increases in activity in the anterior cingulate cortex and orbitofrontal regions of the brain [31].

### 4.1. Radiological Measures

We investigated the alterations in the cross-sectional area and thickness of back muscles in patients experiencing chronic non-specific low back pain after treatment with VRE, IKE, and conventional exercises. The cross-sectional area and thickness of back muscles were gauged using magnetic resonance image and ultrasound, respectively, and an average of the values obtained for these parameters were considered, which shows that these are the reliable method of measurement. Before implementing the intervention program, the baseline data show lower values for cross-sectional area and thickness in the psoas major, quadratus lumborum, multifidus, and erector spinae muscles. This observation is aligns with those of Barker et al., who observed that reductions in the cross-sectional area and muscle thickness are a result of soft tissue injury and inflicted pain, which lead to muscle inhibition. It is the major cause of the reduction in the cross-sectional area of back muscles [32]. In CNLBP, the CSA of the back muscles and back flexors are influencing factors in muscle strength that can affect the core stability of the spine [33]. We observed a statistical improvement in the cross-sectional area of back muscles in the IKE compared with the VRE and conventional exercises. In this trial, the IKE was given at various angular velocities of 60, 90, and 120°/s with maximum rotatory force. Calmes et al. noticed that providing isokinetic exercises at various angular velocities with maximum rotatory force enhanced the back muscle activity and flexor/extensor ratio in sportspersons [18]. These clinical and biomechanical improvements in isokinetic training are in accordance with Lee et al., who further stated that training the back muscles and increasing their strength is the main objective in preventing further consequences in the low back region [34]. The changes noted in the virtual reality training are supported by the prior work conducted by Nambi et al., finding that VRE training protocols facilitate the stimulation of sensory receptors, which increases muscle strength and motor function. VRE training uses real-time feedback to complete the game, and the participants progress quickly to the next stage of the game, which increases the motor activity of the back muscles [35]. In the conventional training exercises, the exercises were targeted to increase the demand for work, which improved motor activity and muscle function. Furthermore, the real physiological and biomechanical changes behind these exercises have not yet been investigated [36].

A recent study by Lee et al. reported that the continuous wear and tear changes at the components of an intervertebral disc and the facet joint are one of the causes of the decrease in the multifidus muscle thickness [37], which can be potentially changed by the existence of fat in the layers of the muscle. The conclusion of this trial was that IKE given at various angular velocities such as 60, 90, and 120°/s with maximum rotatory force could increase the action of muscle tissues at the lower lumbar region, which would result in increased thickness of the multifidus muscles [35].

### 4.2. Inflammatory Biomarker Measures

This trial also compared the changes in inflammatory biomarkers in chronic low back pain patients after virtual, isokinetic, and conventional exercises. The reports show that compared with isokinetic and conventional exercises, virtual reality exercises decrease the inflammation process due to alterations in inflammatory biomarkers such as CRP, TNF-α, and interleukin 2, 4, and 6 levels. Generally, high levels of pro-inflammatory cytokines are present in musculoskeletal and soft tissue injuries. The same inflammatory changes can be seen in chronic non-specific low back pain patients. C-reactive protein and TNF-α levels are controlled by hereditary factors and the presence of fat [38]. In virtual reality training, due to enjoyment, the participants progress to the next level fast and spent more energy than in the other two forms of training. This training consists of high-frequency activities, which could result in changes in these pro-inflammatory cytokines [39]. These exercises also result in secretion of anti-inflammatory cytokines such as antagonists of interleukin-2 and interleukin-4 during VR training [40].

Over four weeks of IKE intervention, the study found an improvement in terms of increased C-reactive protein, tumor necrosis factor-α, and interleukin-6 biomarkers. However, the mechanism behind the positive effect of IKE intervention on pro-inflammatory biomarkers in CNLBP patients is unknown [41]. It has been reported that the application of training at various angular velocities with maximum rotatory force are the cause of changes in the levels of pro-inflammatory cytokines in low back pain. Libardi et al. suggested that the controlled and coordinated exercises provided by the isokinetic device prevent further muscle and joint injuries and enhance tissue healing and repair. These physiological changes are positively associated with the aforementioned inflammatory cytokines [42]. Moreover, regular physical activity and different dietary patterns have a positive influence on inflammatory biomarkers [43,44].

## 5. Strength and Limitations

The real-time appraisal of the radio imaging measures and inflammatory biomarker analysis of different exercise training protocols were the strengths of this research. However, some limitations were noted First, the trial was registered retrospectively, which negates many of the benefits of prospective trial registration, such as preventing non-publication of so called ‘negative’ studies, selective reporting, or changing of primary outcomes. Second, the other primary outcome measures such as muscle strength, functional disability, sleep quality, depression status, and quality of life measures were not assessed. Third, the study did not measure the long-term follow-up effects of these interventions. Lastly, the study did not find a relationship between the radiological and inflammatory changes after VRE and IKE training on chronic non-specific low back pain. The relation between the clinical effects and inflammatory changes resulting due to VRE and IKE needs to be explored in future research to uncover the mechanisms behind the biomechanical and biochemical changes that result in the clinical and inflammatory effects of VRE and IKE in chronic LBP subjects.

## 6. Conclusions

Overall, this investigation showed that exercise with virtual reality and isokinetic decreases pain intensity, increases the muscle cross-sectional area and thickness, and positively alters inflammatory biomarkers compared with conventional training in chronic non-specific low back pain patients. We conclude that both virtual and isokinetic training have equal effects in reducing pain intensity but, at the same time, training through isokinetic training is beneficial in changing radiological measures (muscle cross-sectional area and thickness), and training through virtual reality is beneficial in changing inflammatory measures in soccer players with chronic non-specific low back pain (CNLBP).

## Figures and Tables

**Table 1:** The mean and SD of the demographic characteristics of the VRE, IKE, and conventional groups.

**Sr. No**	**Variable**	**VRE**	**IKE**	**Conventional**	***p*** **-Value**
1	Age (year)	23.2 ± 1.6	22.9 ± 1.7	22.8 ± 1.8	0.742 *
2	Height (meter)	1.72 ± 0.16	1.69 ± 0.17	1.72 ± 0.18	0.813 *
3	Weight (kilogram)	69.5 ± 2.8	70.2 ± 2.4	71.6 ± 2.9	0.051 *
4	BMI (kg/m^2^)	24.5 ± 1.5	23.8 ± 1.4	24.2 ± 1.5	0.325 *
5	maxVO_2_ peak (mL/kg/min)	38.2 ± 4.2	38.6 ± 4.3	37.8 ± 4.3	0.839 *
6	HR (beats/min)	74 ± 6.5	73 ± 6.6	72 ± 7.2	0.648 *
7	Years of playing (year)	5.1 ± 1.3	4.9 ± 1.4	4.8 ± 1.3	0.770 *
8	Duration of Injury (months)	4.8 ± 1.1	5.2 ± 0.9	5.5 ± 0.8	0.070 *

* non-significant, BMI—body mass index, max VO_2_—maximum oxygen uptake, VRE—virtual reality exercise, IKE—isokinetic exercise.

**Table 2:** Mean and SD of pre and post MRI and ultrasound measures of VRE, IKE, and conventional groups.

No	Variable		VRE	IKE	Conventional	F-Score	*p*-Value
1	Pain intensity	Pre	7.2 ± 0.4	7.3 ± 0.3	7.2 ± 0.3	295.60	0.558 *
	VAS (cm)	Post	1.8 ± 0.3	2.5 ± 0.5	4.8 ± 0.4		0.001 **
2	Psoas Major CSA (cm^2^)—R	Pre	8.6 ± 0.4	8.5 ± 0.5	8.5 ± 0.4	102.06	0.705 *
		Post	9.5 ± 0.3	10.1 ± 0.4	8.7 ± 0.2		0.001 **
3	Psoas Major CSA (cm^2^)—L	Pre	7.9 ± 0.5	8.2 ± 0.4	8.1 ± 0.5	170.34	0.271 *
		Post	9.5 ± 0.4	10.2 ± 0.3	8.4 ± 0.2		0.001 **
4	Quadratus. Lumb CSA (cm^2^)—R	Pre	4.6 ± 0.3	4.8 ± 0.4	4.7 ± 0.6	88.82	0.380 *
		Post	5.8 ± 0.3	6.3 ± 0.4	4.9 ± 0.3		0.001 **
5	Quadratus. Lumb CSA (cm^2^)—L	Pre	4.7 ± 0.6	4.5 ± 0.5	4.3 ± 0.5	197.64	0.069 *
		Post	6.5 ± 0.3	6.9 ± 0.4	4.9 ± 0.3		0.001 **
6	Multifidus CSA (cm^2^)—R	Pre	5.6 ± 0.6	5.4 ± 0.6	5.5 ± 0.5	90.87	0.542 *
		Post	7.1 ± 0.5	7.6 ± 0.4	5.8 ± 0.4		0.001 **
7	Multifidus CSA (cm^2^)—L	Pre	5.5 ± 0.5	5.6 ± 0.6	5.6 ± 0.7	71.25	0.834 *
		Post	6.8 ± 0.4	7.4 ± 0.4	5.9 ± 0.4		0.001 **
8	Erector Spinae CSA (cm^2^)—R	Pre	16.2 ± 0.9	15.9 ± 1.3	16.1 ± 1.2	45.97	0.702 *
		Post	17.5 ± 0.5	18.1 ± 0.6	16.4 ± 0.6		0.001 **
9	Erector Spinae CSA (cm^2^)—L	Pre	16.4 ± 1.3	16.5 ± 0.9	16.6 ± 1.2	22.62	0.859 *
		Post	17.7 ± 0.7	18.1 ± 0.5	16.9 ± 0.5		0.001 **
10	Multifidus Thickness (mm)—R	Pre	33.8 ± 3.3	33.6 ± 3.4	32.9 ± 3.6	6.50	0.686 *
		Post	35.2 ± 2.2	35.4 ± 2.3	33.1 ± 2.2		0.002 **
11	Multifidus Thickness (mm)—L	Pre	31.5 ± 2.6	31.6 ± 2.8	32.1 ± 3.2	3.20	0.780 *
		Post	33.5 ± 2.1	34.2 ± 2.1	32.5 ± 2.2		0.047 **

* non-significant, ** significant, VAS—visual analog scale, CSA—cross-sectional area, R—right, L—left, VRE—virtual reality exercise, IKE—isokinetic exercise.

**Table 3:** Mean and SD of pre and post inflammatory biomarker measures of VRE, IKE, and conventional groups.

**Sr. No**	**Variable**		**VRE**	**IKE**	**Conventional**	**F-Score**	***p*** **-Value**
1	CRP mg/L	Pre	1.56 ± 0.3	1.58 ± 0.4	1.57 ± 0.4	818.79	0.985 *
		Post	0.4 ± 0.08	0.9 ± 0.07	1.3 ± 0.06		0.001 **
2	TNF-α pg/mL	Pre	16.6 ± 0.7	16.2 ± 0.5	16.5 ± 0.6	1017.44	0.103 *
		Post	7.7 ± 0.5	10.2 ± 0.6	15.2 ± 0.5		0.001 **
3	IL-2 pg/mL	Pre	12.3 ± 0.5	12.1 ± 0.6	12.3 ± 0.4	208.83	0.360 *
		Post	15.8 ± 0.5	14.8 ± 0.6	12.6 ± 0.4		0.001 **
4	IL-4 pg/mL	Pre	39.5 ± 3.6	39.3 ± 3.7	39.2 ± 3.6	103.55	0.965 *
		Post	65.3 ± 5.6	59.2 ± 4.9	44.3 ± 3.5		0.001 **
5	IL-6 pg/mL	Pre	5.5 ± 0.5	5.4 ± 0.4	5.7 ± 0.6	329.75	0.171 *
		Post	1.9 ± 0.3	3.5 ± 0.4	4.9 ± 0.4		0.001 **

* non-significant, ** significant, CRP—C-reactive protein, TNF-α—tumor necrosing factor, IL—interleukin, VRE—virtual reality exercise, IKE—isokinetic exercise.

**Figure A1:**
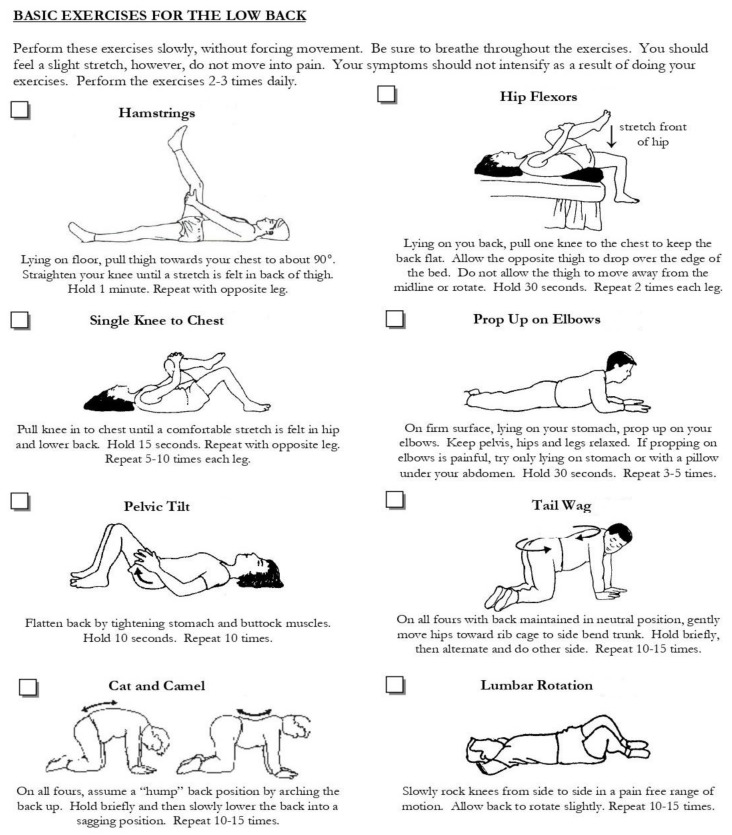
Basic exercises for the low back region.

## Data Availability

Data are not publicly available but can be obtained from the corresponding author on request.
